# Reference Values of Peripheral Perfusion Index in Healthy Neonates and Preliminary Observations of Congenital Heart Disease in Tibet: A Cohort Study

**DOI:** 10.3390/ijns12030054

**Published:** 2026-07-20

**Authors:** Miaomiao Wei, Yaoyao Dong, Ciren Zhuoma, Lin Peng, Youping Tian, Qing Gu, Sheng Wei, Bianba Zhuoma, Xiaojing Hu, Guoying Huang

**Affiliations:** 1 Lhasa People’s Hospital, Lhasa 850000, China; 2 Neonatal Intensive Care Unit, Children’s Hospital of Fudan University, Shanghai 200000, China; 3 School of Nursing, Fudan University, Shanghai 200000, China; 4 National Management Office of Neonatal Screening Program for CHD, Shanghai 200000, China; 5 Shanghai Key Laboratory of Birth Defects, Shanghai 200000, China; 6 Department of Nursing, Children’s Hospital of Fudan University, Shanghai 200000, China; 7 Fujian Key Laboratory of Neonatal Diseases, Xiamen 361000, China

**Keywords:** peripheral perfusion index, neonate, high altitude, congenital heart disease, reference values

## Abstract

The peripheral perfusion index (PPI) is a non-invasive indicator of peripheral tissue perfusion in neonates. However, reference values for the PPI at different postnatal time points in healthy neonates residing at high altitudes, as well as early perfusion characteristics in infants with congenital heart disease (CHD) in these settings, remain inadequately defined. The primary aim was to determine the dynamic changes in and percentile-based reference values of the PPI in healthy neonates during the first 6–72 h after birth in the high-altitude region of Tibet. A secondary, exploratory aim was to observe PPI patterns in neonates with CHD born during the same period and to compare the two groups, given the limited number of CHD cases enrolled. A longitudinal cohort study was conducted among consecutively born neonates in a high-altitude region of Tibet. The PPI was measured in the right hand (pre-ductal) and either foot (post-ductal) at 6, ~12, ~24, ~48, and ~72 h after birth. Generalized Additive Models for Location, Scale and Shape (GAMLSS) were used to construct percentile reference curves for healthy neonates. Generalized Estimating Equations (GEE) were applied to evaluate differences in the PPI across time points, measurement sites, and between groups (healthy vs. CHD). A total of 1043 neonates were enrolled, including 1034 in the healthy group and only 9 CHD cases. In healthy neonates, the PPI increased progressively within the first 72 h, with a rapid rise from 6 to 24 h, a slower increase from 24 to 48 h, and stabilization between 48 and 72 h. At all time points, the lower-limb PPI was significantly higher than the upper-limb PPI (*p* < 0.001). The 50th percentile (P_50_) values for the upper-limb PPI at 6, 12, 24, 48, and 72 h were 2.32, 2.46, 2.62, 2.63, and 2.77, respectively; corresponding values for the lower-limb PPI were 2.53, 2.64, 2.76, 2.77, and 2.90. Compared to the healthy group, neonates with CHD at high altitudes had similar PPI at 6 h. However, their PPI was significantly lower between 12 and 48 h. At 72 h, no statistically significant difference was found between the groups, likely due to the small sample size of CHD infants. Conclusions: This study provides a reference for routine perfusion assessment in stable neonates born at high altitudes. Due to the limited sample size (only 9 CHD cases), our findings on PPI changes in neonates with CHD remain preliminary. The normative PPI percentile curves established in this study offer objective baseline perfusion data for stable neonates living in high-altitude areas. Future large-scale cohort studies are still needed to validate the clinical value of the PPI as a perfusion monitoring tool in infants with CHD.

## 1. Background

Congenital heart disease (CHD) is the most prevalent birth defect in newborns [1], with an estimated incidence ranging from 8 to 10 per 1000 live births [2,3,4]. Critical congenital heart disease (CCHD), which comprises approximately 25% of all CHD cases, can lead to neonatal shock, severe hypoxia, and even death if not diagnosed and treated promptly [5]. Early detection is therefore crucial for initiating timely interventions and improving outcomes in affected infants.

The peripheral perfusion index (PPI), a non-invasive parameter derived from pulse oximetry, reflects the ratio of pulsatile to non-pulsatile blood flow in peripheral tissues and serves as an indirect measure of peripheral circulatory perfusion [6]. Several studies have demonstrated the utility of the PPI as an adjunct screening tool for CHD, particularly for left-sided obstructive lesions [7]. However, PPI values are influenced by various factors, including altitude, postnatal age, and measurement site [8,9,10]. Based on the detection principle of the PPI, cardiac output is its key influencing factor. Severe hypoxemia at high altitudes forces the heart to increase blood supply to satisfy systemic oxygen demand, which may increase neonatal PPI levels [10]. To date, limited data are available on PPI values in high-altitude regions such as Tibet (≈3658 m above sea level).

Generalized additive models for location, scale, and shape (GAMLSS) provide a flexible semi-parametric framework for modeling response variables that do not necessarily follow an exponential family distribution [11,12,13]. This approach is particularly suitable for fitting skewed or kurtotic data and has been widely used in constructing pediatric growth and weight reference curves [14].

In this longitudinal cohort study, the primary objective was to apply the GAMLSS to establish dynamic percentile reference curves for the PPI in healthy Tibetan newborns at 6, ~12, ~24, ~48, and ~72 h after birth in a high-altitude setting. We also evaluated the influence of measurement site (right upper limb vs. lower limb) on PPI values. As a secondary exploratory analysis, this study examined the early PPI profiles of neonates diagnosed with CHD within the first 72 h after birth and conducted a comparative analysis against the corresponding profiles of healthy neonates. We aimed to establish normative PPI baselines for neonates born at plateau altitudes, while the limited CHD subgroup was analyzed to offer preliminary clues for subsequent relevant research.

## 2. Materials and Methods

### 2.1. Study Design and Participants

This study used a prospective cohort design with a post hoc analytical approach. The study consisted of two parallel arms: a Healthy Neonates Cohort, intended to establish normative PPI reference curves, and a CHD Cohort, designed to longitudinally assess PPI values and compare them with those of healthy controls.

Initial Enrollment (Broad Criteria): Participants were consecutively delivered newborns at Lhasa People’s Hospital between February 2025 and December 2025, including both healthy neonates and those diagnosed with CHD. To minimize selection bias, no exclusions were applied at the time of enrollment.

#### 2.1.1. Post Hoc Group Definitions

(1) Healthy Neonates (Reference Group):

Inclusion criteria: Consecutively delivered newborns at the study hospital; No requirement for supplemental oxygen within the first 72 h after birth; Gestational age ≥ 34 weeks; Maternal residence in the local area throughout pregnancy; Voluntary participation with signed informed consent from parents.

Exclusion criteria: Neonates without CHD who had an Apgar score below 6; Neonates requiring respiratory support; Neonates born at a gestational age of less than 34 weeks; Neonates presenting with clinical or hemodynamic instability; Neonates who died or were transferred to other departments.

(2) CHD Infants (Case Group):

Inclusion criteria: Echocardiographically confirmed CHD (e.g., VSD, ASD, TOF).

Exclusion criteria: Concurrent arrhythmias (e.g., complete heart block, atrial flutter); Requirement for mechanical circulatory support or pacemaker dependence; Major non-cardiac anomalies (e.g., genetic syndromes).

The following echocardiographic findings were not classified as definite CHD in this study: ① PDA lesions that spontaneously closed within 3 months after delivery; ② Isolated ASD with shunt width less than 5 mm upon 3-month follow-up; ③ Transient physiological acceleration of blood flow in bilateral pulmonary artery branches; ④ Stable pulmonary stenosis (PS) and aortic stenosis (AS) with peak transvalvular pressure gradients below 20 mmHg without progressive deterioration; ⑤ Pure isolated patent foramen ovale (PFO), and bicuspid aortic valve without concomitant stenosis or substantial valvular regurgitation.

#### 2.1.2. Ethical Considerations

The study was approved by the Ethics Committee of Lhasa People’s Hospital (Approval No. SYLL2224087). Written informed consent was obtained from the parents or guardians of all participating neonates.

#### 2.1.3. Sample Size Calculation

A sufficiently large sample size is essential for accurately estimating extreme percentiles, such as the 2.5th percentile. In this study, the precision level (defined as the allowable error, *D*) was set at 0.1 times the standard deviation (*SD*), with a two-tailed test at a significance level (*α*) of 0.05. The initial sample size was calculated as 385 neonates using R software (version 4.4.0). To account for potential sampling errors and participant attrition, a 30% increase was applied, resulting in a final required sample size of approximately 500 neonates. During continuous enrollment, a larger number of eligible healthy neonates meeting all inclusion criteria were available; we consecutively recruited all qualified infants and obtained a final sample of 1079 participants. This expanded sample substantially enhanced the statistical precision of the extreme tail percentiles of the PPI reference ranges.

$$n={\left(\frac{Z_{\alpha /2}\times SD}{D}\right)}^{2}$$

### 2.2. Procedures

Baseline neonatal data were collected by trained personnel who had completed standardized training and obtained certification for neonatal CHD screening. Prior to project implementation, all data collectors and data entry staff involved in this study received unified specialized training covering PPI operation standards and echocardiography screening protocols; only those who passed theoretical and practical assessment were permitted to perform data collection and entry. All participants received universal postnatal echocardiographic screening.

Demographic and clinical data were extracted from the hospital medical record system, including gender, ethnicity, gestational age (GA), birth weight, mode of delivery, Apgar score, and other relevant clinical indicators.

PPI measurements were performed at 6, 12, 24, 48, and 72 h after birth (±1 h). For neonates discharged before the 72 h time point, the final measurement was taken before discharge. The right hand (RH) was designated as the pre-ductal measurement site, and either foot was selected as the post-ductal measurement site, to ensure consistent limb selection across all study neonates. All measurements were conducted with the infant in a supine position and in a calm awake or sleeping state, excluding periods of feeding, crying, or physical discomfort. Opaque ambient light shields were placed around the probe to avoid extraneous light exposure, all PPI measurements were performed under strictly standardized ward temperature and humidity conditions, with negligible environmental differences among individual neonates. After the PPI waveform signals reached a stable state, three consecutive readings were recorded at 1 min intervals for each time point, and the average value of these readings was adopted for subsequent statistical analysis. Correct probe placement was standardized to prevent optical shunting during detection.

The Masimo RADIC-7 motion-resistant pulse oximeter (Masimo, Irvine, CA, USA) with reusable LNCS Y1 multi-site foam probes was used for PPI detection. The probes were attached to the right hand and the sole of either foot with foam wraps. PPI values were recorded only after stable readings were maintained for at least 6 s. Heart rate and body temperature were simultaneously documented during each measurement. Data entry staff accurately recorded relevant information, including measurement date, postnatal age (in hours), and infant state (awake or asleep).

### 2.3. Statistical Analysis

All statistical analyses were conducted using R software (version 4.3.2). Normally distributed continuous variables were presented as mean ± standard deviation, while non-normally distributed continuous variables were expressed as median and interquartile range [M (P25, P75)]. Categorical variables were reported as frequencies and percentages [*n* (%)]. For continuous variables, comparisons between the healthy group and the CHD group were performed using the independent-samples *t*-test for data with normal distribution and homogeneous variance, and the Welch’s corrected *t*-test was applied for non-normally distributed data. The Pearson chi-square test was used for comparisons of categorical variables. The GAMLSS was established to construct the distribution curves of PPI values in healthy neonates. Missing data were imputed using the multiple imputation (MI) method via the mice package in R. We reported the proportion of missing data, all covariates included in the imputation model; PPI outcome values were not imputed in this study. To fully capture the complex distribution characteristics of the PPI, multiple distribution families, including BCCG, BCPE, BCT, and normal distribution, were evaluated. The optimal model was selected based on the Generalized Akaike Information Criterion (GAIC). Percentile reference curves and corresponding percentile values of the PPI at 6, 12, 24, 48, and 72 h after birth were then established for healthy neonates residing in high-altitude areas. Neonates were stratified into late preterm (34–36+6 weeks) and term (≥37 weeks) subgroups to conduct stratified analyses of PPI reference values.

Generalized estimating equations (GEE) were employed to analyze repeated measurement data. ① For healthy neonates, a GEE model was constructed to examine the changing trends in the PPI in relation to postnatal time (with 72 h as the reference) and measurement site (with the lower limb as the reference), incorporating a time-site interaction term. ② To evaluate dynamic intergroup differences between the CHD group and the healthy group, another GEE model was established, adjusting for the group-time interaction term. Given the limited sample size of only nine CHD infants, all GEE-derived effect estimates for the CHD subgroup were interpreted with extreme caution, and we prioritized descriptive analysis of individual PPI trajectories, value ranges of CHD cases. GEE accounts for the intra-correlation inherent in repeated measurements and provides unbiased estimates even in the presence of missing data [15]. All statistical tests were two-tailed, and a *p* value of less than 0.05 was considered statistically significant.

## 3. Results

### 3.1. Baseline Characteristics of Study Participants

**Figure 1:**
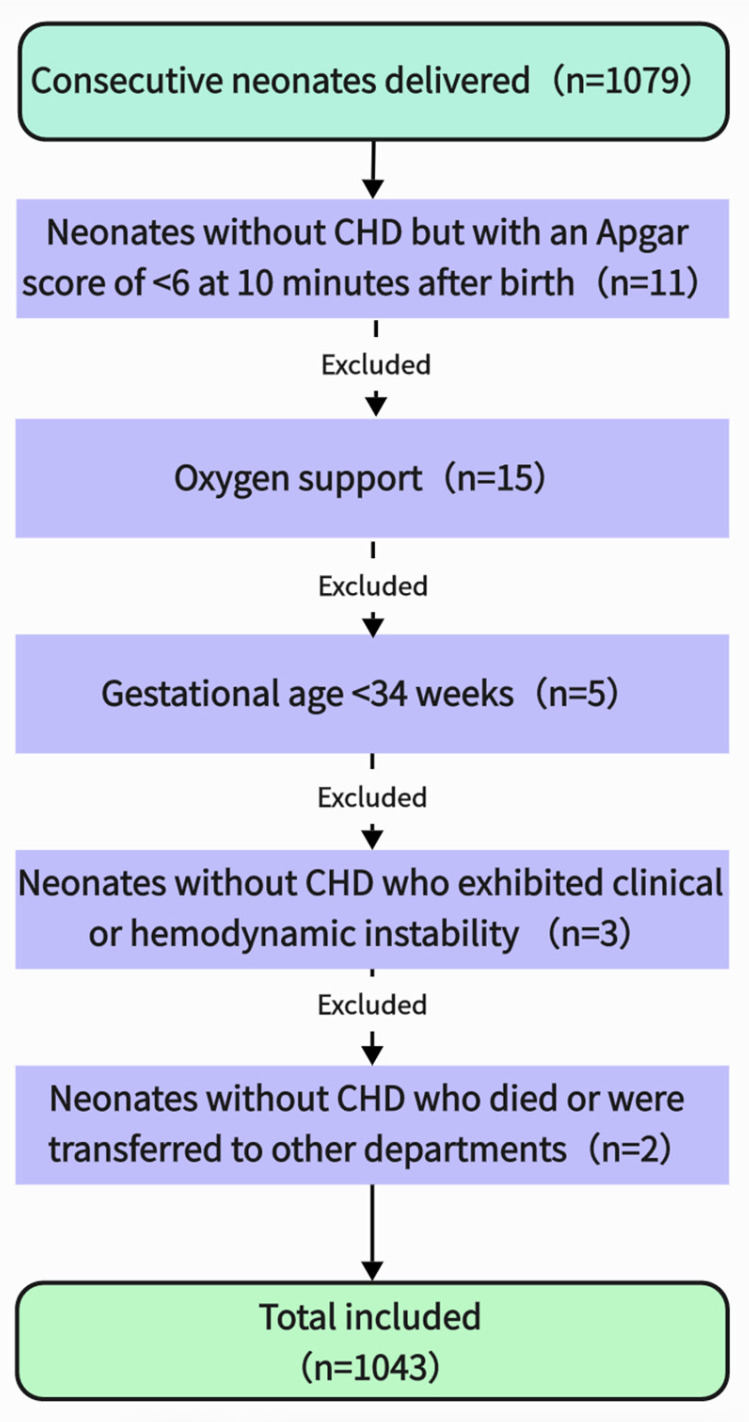
Flowchart of Participant Inclusion and Exclusion.

During the study period, a total of 1079 neonates were born. Following the application of the inclusion and exclusion criteria, neonates without CHD who had an Apgar score below 6 (*n* = 11), those requiring respiratory support (*n* = 15), neonates born at a gestational age of less than 34 weeks (*n* = 5), those presenting with clinical or hemodynamic instability (*n* = 3), and neonates who died or were transferred to other departments (*n* = 2) were excluded. Ultimately, 1043 neonates successfully completed PPI measurement and were included in the final analysis (Figure 1). All enrolled participants were divided into a healthy group (*N* = 1034) and a CHD group (*N* = 9) based on the diagnosis of congenital heart disease (Table 1).

In both groups, the majority of participants were of Tibetan ethnicity and from singleton pregnancies, and none of the mothers reported a history of smoking. No significant differences were observed between the two groups regarding gender, mode of delivery, ethnicity, or singleton status. For continuous variables, there were no statistically significant differences between the groups in gestational age; birth weight; 1, 5, and 10 min Apgar scores; heart rate; body temperature at 6 h after birth; length of hospital stay; or pre-ductal and post-ductal PPI values at 6 h postpartum (Table 1).

Missing data rates for the serial PPI at 6, 12, 24, 48, and 72 h were 8%, 11%, 12%, 14%, and 15%; 1/5/10 min Apgar scores had missing rates of 2%, 3%, and 4%. Missing proportions for heart rate and ethnicity were 1% and 0.35%, respectively. All variables had missingness <30%, meeting the criteria for multiple imputation.

### 3.2. Establishment of Reference Values for PPI in Healthy Neonates

#### 3.2.1. Effects of Postnatal Time and Measurement Site on PPI in Healthy Neonates Based on the GEE Model

Based on the GEE model, a significant main effect of postnatal time on the PPI was observed. Compared with the 72 h time point, PPI values gradually increased over time, with the lowest value recorded at 6 h and the highest at 72 h, and the differences were statistically significant (*p* < 0.001). Measurement site also influenced PPI values, with the lower limb PPI being significantly higher than the upper limb PPI (β = −0.18, *p* < 0.001). A significant interaction between time and measurement site was identified (*p* < 0.001), indicating that the difference in the PPI between the upper and lower limbs at 6 h after birth was greater than that at other time points. Gestational age had no significant effect on the PPI (*p* = 0.225) (Table 2).

#### 3.2.2. Percentile Values and Dynamic Trajectory of PPI in Healthy Neonates Within 6–72 h After Birth

The GAMLSS was used to establish percentile reference ranges for PPI values in healthy neonates during the first 72 h after birth, as summarized in Table 3. At 6 h postnatally, the median (P_50_) upper limb PPI was 2.32 (P_2.5_–P_97.5_: 0.55–4.15). As postnatal age increased, the P_50_ of the upper limb PPI sequentially rose to 2.46 (0.67–4.32), 2.62 (0.92–4.40), 2.63 (1.32–4.04), and 2.77 (1.70–3.96) at 12, 24, 48, and 72 h, respectively. Lower limb PPI values were significantly higher than those of the upper limb at all time points (*p* < 0.001). The corresponding P_50_ values for the lower limb PPI were 2.53 (0.75–4.53), 2.64 (0.89–4.63), 2.76 (1.13–4.65), 2.77 (1.48–4.31), and 2.90 (1.83–4.21).

**Figure 2:**
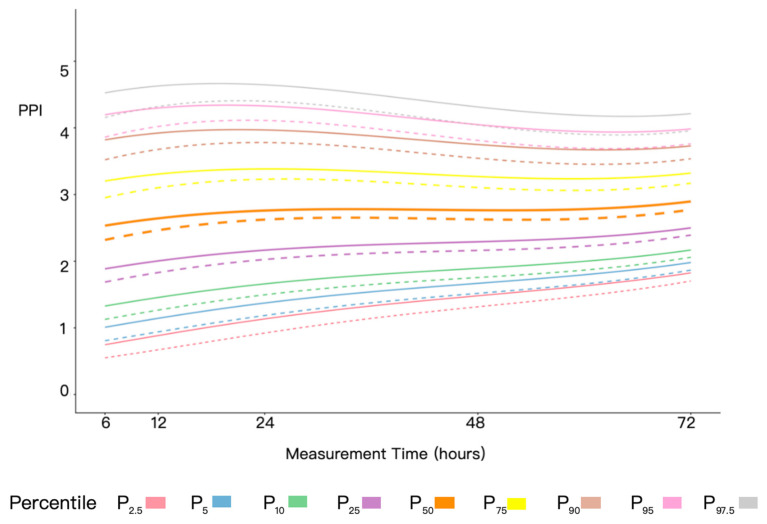
Percentile distribution curve of PPI in healthy neonates within 6 to 72 h after birth (Note: solid lines represent the upper limb, and dashed lines represent the lower limb).

PPI values for both upper and lower limbs presented consistent dynamic trends over time, as illustrated in Figure 2. PPI levels increased rapidly from 6 to 24 h, followed by a modest change between 24 and 48 h, and eventually stabilized during the 48- to 72 h period. The inter-percentile range (P_97.5_–P_2.5_) at 6 h was 3.60 for the upper limb and 3.78 for the lower limb; these ranges narrowed to 2.26 and 2.38 at 72 h, respectively. These findings suggest that the distribution of PPI values became progressively more concentrated with advancing postnatal age.

#### 3.2.3. Gestational Age-Stratified Percentile Characteristics and Trajectories of Neonatal PPI Within 6–72 h After Birth

**Figure 3:**
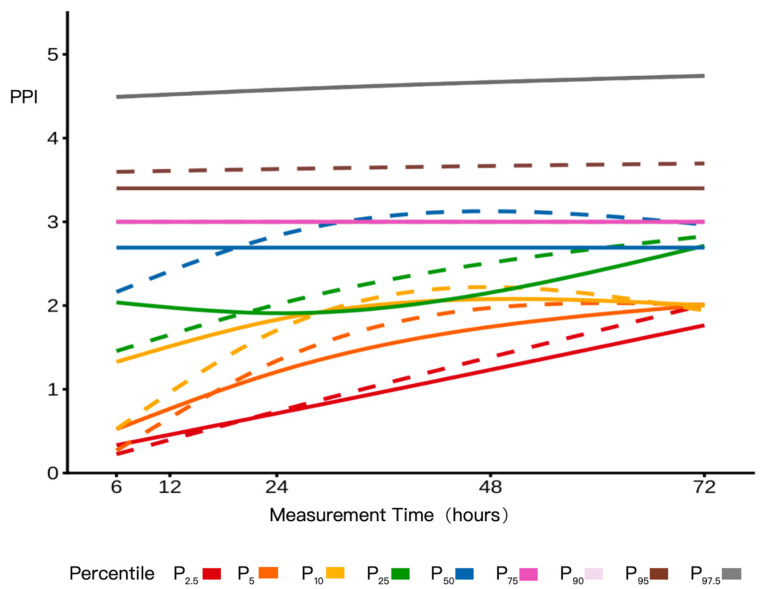
Gestational age-stratified percentile distribution curves of PPI in healthy neonates within 6 to 72 h after birth (Note: solid lines represent the upper limb, and dashed lines represent the lower limb; The P90 curve fully coincides with the P75 curve across all time points).

GAMLSS generated separate PPI percentile references for late preterm and term neonates (Table 4, Figure 3). The median PPI increased from 2.16 to 2.97 over 6–72 h in late preterm infants, while the median stayed at 2.69 in term infants. PPI values rose with postnatal age in both groups. Their percentile curves mostly overlapped; minor gaps only existed in low percentiles at early time points, and high percentiles were nearly identical between subgroups.

#### 3.2.4. Preliminary Exploratory Analysis of Early Perfusion Characteristics in Neonates with CHD

This CHD subgroup analysis is exploratory and limited to only 9 infants; all intergroup comparisons are preliminary observations. Full clinical details of these CHD cases are presented in Table 5. Among the 9 identified neonates with CHD, patent ductus arteriosus (PDA) was the most common subtype (5 cases), followed by ventricular septal defect (VSD, 3 cases) and atrial septal defect (ASD, 1 case). This CHD subgroup analysis is exploratory and limited to only 9 infants; all intergroup comparisons are preliminary observations. All PDAs showed a measurable ductal diameter and left-to-right shunting on echocardiography. They remained persistently patent beyond three months of clinical follow-up. These findings meet established criteria for hemodynamically significant patent ductus arteriosus. This distinguishes them from transient physiological ductal patency commonly seen in early neonatal adaptation.

#### 3.2.5. Comparison of PPI Levels Between Healthy and CHD Neonates at Multiple Postnatal Time Points

The GEE model was used to compare PPI values between the healthy and CHD groups across different postnatal time points. In the healthy group, PPI levels demonstrated a continuous upward trend with increasing postnatal age, rising from 2.25 ± 0.03 at 6 h to 2.79 ± 0.03 at 72 h. At 6 h postpartum, the CHD group demonstrated slightly higher PPI values than the healthy group, although the difference was not statistically significant (*p* = 0.0996). In contrast, CHD infants showed significantly lower PPI values at 12, 24, and 48 h compared with healthy neonates (*p* < 0.05). At 72 h after birth, PPI levels in the CHD group (2.46 ± 0.09) remained lower than those in the healthy group (2.79 ± 0.03), but the difference did not reach statistical significance (*p* = 0.0706). A detailed comparison of PPI values between the two groups at each postnatal time point is presented in Table 6.

## 4. Discussion

### 4.1. Clinical Implications of Percentile Reference Standards for Neonatal PPI Within 6–72 h After Birth

This study established percentile-based reference ranges (P_2.5_–P_97.5_) for the PPI in the upper and lower limbs of healthy neonates at an altitude of 3650 m, specifically at 6, 12, 24, 48, and 72 h after birth. The median PPI values observed in this high-altitude cohort (upper limb: 2.32 to 2.77; lower limb: 2.53 to 2.90) were consistent with findings from similar high-altitude settings [10] but were notably higher than those reported for neonates at sea level [7,16] and moderate altitudes [17]. This pattern underscores an association between increasing altitude and elevated neonatal PPI values. The underlying physiological mechanism likely involves compensatory hemodynamic adjustments to chronic hypoxia. As altitude rises, the concomitant decrease in atmospheric pressure and arterial oxygen saturation [18,19,20] necessitates increased cardiac output to maintain tissue oxygen delivery, which is reflected in higher PPI values. This altitude-dependent variation has critical clinical implications. While a PPI value ≤ 1.24 has been validated as a predictor of illness severity at sea level [21], our findings suggest that this diagnostic threshold requires upward adjustment for neonates residing in high-altitude regions due to their physiologically elevated baseline PPI.

### 4.2. Dynamic Change and Measurement Site Variation in Neonatal PPI

A key finding of this study was the dynamic trajectory of the PPI in healthy neonates during the first 72 h of life. PPI values demonstrated a progressive increase, characterized by a rapid rise from 6 to 24 h, a slower ascent from 24 to 48 h, and subsequent stabilization from 48 to 72 h. This pattern, also observed by Hakan et al. [16], aligns with the physiological transition from fetal to neonatal circulation. The rapid decrease in pulmonary vascular resistance and the corresponding increase in systemic blood flow during this period lead to a gradual improvement in peripheral perfusion, culminating in the observed stabilization of the PPI. Furthermore, the narrowing range between the P_97.5_ and P_2.5_ percentiles over time indicates a reduction in inter-individual variability as neonates adapt to extrauterine life. The study also identified a statistically significant difference in PPI values between measurement sites, with lower limb values consistently exceeding those of the upper limb (β = −0.14, *p* < 0.001). This discrepancy was most pronounced at the 6 h mark. While consistent with some reports [9], it contrasts with others [8,16]. A plausible explanation for our observation is the influence of local temperature, as the infants’ feet were typically wrapped, potentially preserving higher local temperature and perfusion compared to the exposed hands. Given that the PPI is sensitive to temperature-induced vasoconstriction or vasodilation [22], these findings highlight the importance of standardizing measurement conditions, including site, postnatal timing, and thermal management, in clinical practice.

### 4.3. Alterations in PPI Among Neonates with CHD

The PPI trajectory in neonates with CHD exhibited distinct temporal patterns compared to healthy controls. At 6 h postpartum, PPI values in the CHD group were slightly elevated, though not significantly, possibly reflecting a non-specific physiological stress response to birth [23]. Subsequently, from 12 to 48 h, the CHD group displayed significantly lower PPI values (*p* < 0.05). Given the extremely limited sample of only 9 CHD infants, these intergroup differences should be regarded as preliminary exploratory observations. This reduction in peripheral perfusion may partially relate to the pathophysiology of CHD. The activation of neurohormonal systems (e.g., renin-angiotensin-aldosterone and sympathetic nervous systems) increases systemic vascular resistance [24], while hypoxia-driven mechanisms—such as the upregulation of vascular endothelial growth factor (VEGF) and reduced prostaglandin E2 degradation—promote pulmonary vasodilation and ductal patency [25]. Compared with prior reports, hemodynamically significant PDA leads to a lower postductal foot PPI relative to the preductal right-hand PPI, as left-to-right ductal shunting diminishes descending aortic perfusion [26]. These changes collectively cause a left-to-right shunt, diverting a significant portion of cardiac output to the pulmonary circulation at the expense of systemic perfusion, thereby lowering PPI values [27]. However, our sample size is limited, and larger cohorts are needed to verify these preliminary perfusion gradient observations. At 72 h, PPI values were still numerically lower in CHD neonates, and the non-significant intergroup difference was likely caused by the small sample size.

### 4.4. Limitations

This study has several limitations. The exclusion of neonates with a gestational age below 34 weeks and low birth weight means the established reference standards may not be applicable to these vulnerable populations, warranting further investigation. Additionally, as the study population was predominantly Tibetan, the generalizability of these reference values to Han Chinese neonates residing at high altitudes remains uncertain due to the limited number of Han participants.

All unstable neonates requiring respiratory support or hemodynamic intervention were excluded from the reference cohort, which restricts the generalizability of our PPI reference curves only to clinically stable high-altitude newborns.

Only nine neonates with CHD were enrolled in the present study. Due to the small sample size and the predominance of mild lesions (mostly PDA/VSD/ASD), all intergroup comparisons of PPI values between CHD infants and healthy neonates should be interpreted as exploratory findings rather than definitive conclusions. Most CHD cases presented mild left-to-right shunt lesions; therefore, these results cannot be extended to neonates with severe complex congenital heart defects. Moreover, GEE-derived effect estimates for the CHD subgroup are likely unstable and require cautious interpretation. This single-center cohort only characterizes neonatal PPI profiles at a fixed high-altitude site (~3700 m) in Tibet, so our findings cannot establish causal relationships between altitude and the PPI across a range of elevation gradients.

## 5. Conclusions

This study established the percentile reference standards for the PPI of the upper and lower limbs in healthy neonates at 6, 12, 24, 48, and 72 h after birth in the high-altitude region of Tibet. The PPI values of healthy neonates gradually increased within the first 6 to 72 h after birth and subsequently stabilized. PPI values in the lower limb were consistently higher than those in the upper limb, with the most pronounced difference between measurement sites observed at 6 h after birth. In the early postnatal period (6 h), no significant difference in PPI values was found between neonates with CHD and healthy neonates. However, from 12 to 48 h after birth, PPI values in the CHD group were significantly lower than those in the healthy group, yet no significant intergroup difference was present at 72 h. This lack of statistical significance at 72 h is likely attributable to limited sample size. The primary contribution of this study lies in the high-altitude-specific percentile PPI reference curves for clinically stable healthy neonates, which can support routine perfusion evaluation in local neonatal care. CHD-related PPI findings remain exploratory, and larger cohorts are needed to verify its clinical predictive performance.

## Figures and Tables

**Table 1:** Baseline Characteristics of Participants at Six Hours After Birth.

Characteristic	Healthy Neonate Group (*N* = 1034)	Congenital Heart Disease Group (*N* = 9)	Total (*N* = 1043)	*p* Value
**Ethnicity, *n*** **(%)**				1.000
Han nationality	7 (0.7)	0 (0.0)	7 (0.7)	
Tibetan nationality	1027 (99.3)	9 (100.0)	1036 (99.3)	
**Mothers with no smoking history, *n*** **(%)**	1034 (100.0)	9 (100.0)	1043 (100.0)	NA
**Gender, *n*** **(%)**				0.374
Female	478 (46.2)	6 (66.7)	484 (46.4)	
Male	556 (53.8)	3 (33.3)	559 (53.6)	
**Gestational age, mean ± SD** **(week)**	39.31 ± 1.36	39.89 ± 1.36	39.32 ± 1.36	0.205
**Birth weight, mean ± SD** **(g)**	3149.79 ± 450.81	3383.89 ± 371.24	3154.12 ± 448.97	0.121
**Apgar score, mean ± SD**				
1 min	9.98 ± 0.17	10.00 ± 0.00	9.98 ± 0.17	0.706
5 min	9.99 ± 0.14	10.00 ± 0.00	9.99 ± 0.14	0.881
10 min	10.00 ± 0.00	10.00 ± 0.00	10.00 ± 0.00	NA
**Mode of delivery, *n*** **(%)**				0.191
Cesarean section	309 (29.9)	5 (55.6)	314 (30.1)	
Vaginal delivery	725 (70.1)	4 (44.4)	729 (69.9)	
**Singleton pregnancy, *n*** **(%)**				1.000
No	18 (1.7)	0 (0.0)	18 (1.7)	
Yes	1016 (98.3)	9 (100.0)	1025 (98.3)	
**Heart rate, mean ± SD** **(beats/min)**	137.66 ± 8.38	135.89 ± 11.21	137.61 ± 8.45	0.530
**Body temperature, mean ± SD (°C)**	36.42 ± 0.18	36.49 ± 0.14	36.42 ± 0.18	0.299
**Hospital stay, mean ± SD (days)**	5.46 ± 4.13	7.67 ± 4.30	5.49 ± 4.14	0.111
**Pre-ductal PPI, mean ± SD (%)**	2.25 ± 0.94	2.61 ± 0.70	2.26 ± 0.94	0.254
**Post-ductal PPI, mean ± SD (%)**	2.50 ± 1.01	2.11 ± 0.89	2.49 ± 1.01	0.247

**Table 2:** Changes in PPI Values of Healthy Neonates According to Postnatal Time, Measurement Site and Gestational Age.

Parameter^①^	β (95% CI)	*p* Value
**Intercept**	3.05 (2.87–3.23)	<0.001
**Time, hour (reference: 72 h)** **^②^**		
6 h	−0.48 (−0.53–−0.43)	0.001
12 h	−0.22 (−0.27–−0.17)	<0.001
24 h	−0.14 (−0.18–−0.10)	<0.001
48 h	−0.13 (−0.17–−0.10)	<0.001
**Measurement site (reference: lower limb)** **^②^**		
Upper limb	−0.18 (−0.22–−0.13)	<0.001
**Time × measurement site interaction** **^②^**		
6 h × upper limb	−0.11 (−0.21–−0.02)	<0.05
**Gestational age (reference: late preterm)** **^②^**		
Term	−0.11 (−0.28–0.07)	0.225

^①^ GEE were used to evaluate the effects of time, measurement site, their interaction and gestational age, with β estimates, 95% confidence intervals (CIs), and *p*-values reported. ^②^ Reference groups: time = 72 h; measurement site = lower limb; gestational age = late preterm (34–36+6 weeks).

**Table 3:** Percentile Reference Standards of PPI Curves in Healthy Neonates Within 6–72 h After Birth.

Measurement Site	Time Point (h)	P_2.5_	P_5_	P_10_	P_25_	P_50_	P_75_	P_90_	P_95_	P_97.5_
Upper Limb	6	0.55	0.81	1.13	1.69	2.32	2.95	3.52	3.86	4.15
	12	0.67	0.94	1.27	1.83	2.46	3.10	3.68	4.02	4.32
	24	0.92	1.19	1.50	2.03	2.62	3.23	3.78	4.11	4.40
	48	1.32	1.52	1.76	2.16	2.63	3.11	3.54	3.81	4.04
	72	1.70	1.87	2.06	2.39	2.77	3.17	3.54	3.76	3.96
Lower Limb	6	0.75	1.01	1.33	1.89	2.53	3.20	3.82	4.20	4.53
	12	0.89	1.14	1.46	2.00	2.64	3.31	3.92	4.30	4.63
	24	1.13	1.37	1.66	2.17	2.76	3.38	3.97	4.33	4.65
	48	1.48	1.67	1.89	2.29	2.77	3.27	3.75	4.05	4.31
	72	1.83	1.98	2.17	2.50	2.90	3.32	3.73	3.98	4.21

**Table 4:** Gestational Age-Stratified Percentile Reference Standards of PPI in Healthy Neonates Within 6–72 h After Birth.

Gestational Age	Time Point (h)	P_2.5_	P_5_	P_10_	P_25_	P_50_	P_75_	P_90_	P_95_	P_97.5_
Late preterm	6	0.23	0.27	0.52	1.46	2.16	3.00	3.00	3.60	4.49
	12	0.40	0.66	0.96	1.65	2.41	3.00	3.00	3.61	4.52
	24	0.74	1.34	1.71	2.01	2.83	3.00	3.00	3.63	4.58
	48	1.38	1.97	2.22	2.51	3.13	3.00	3.00	3.67	4.67
	72	2.01	2.01	1.94	2.83	2.97	3.00	3.00	3.70	4.74
Term	6	0.33	0.52	1.33	2.04	2.69	3.00	3.00	3.40	4.49
	12	0.46	0.77	1.51	1.98	2.69	3.00	3.00	3.40	4.52
	24	0.71	1.21	1.83	1.91	2.69	3.00	3.00	3.40	4.58
	48	1.23	1.75	2.08	2.16	2.69	3.00	3.00	3.40	4.67
	72	1.76	2.00	2.00	2.71	2.69	3.00	3.00	3.40	4.74

**Table 5:** Echocardiographic diagnoses and serial preductal/postductal PPI of nine CHD neonates.

Case No.	Cardiac Lesions	PPI—Upper Limb (6/12/24/48/72 h)	PPI—Lower Limb (6/12/24/48/72 h)
1	Muscular VSD 0.19 cm	3.00, 2.00, 2.63, 2.67, 2.79	2.00, 2.00, 2.79, 2.81, 2.93
2	VSD 0.3 cm	3.00, 2.50, 3.00, 2.50, 2.00	2.50, 3.00, 2.79, 3.00, 2.00
3	VSD	3.00, 2.00, 3.00, 2.00, 3.00	2.00, 3.00, 2.00, 2.00, 3.00
4	ASD 0.5 cm	3.00, 2.00, 2.00, 3.00, 3.00	3.00, 2.00, 2.00, 3.00, 3.00
5	PDA 0.45 cm	3.00, 2.00, 3.00, 2.00, 1.75	2.00, 3.00, 3.00, 3.00, 2.00
6	PDA 0.51 cm	2.00, 2.00, 2.00, 3.00, 3.00	1.00, 2.00, 3.00, 3.00, 3.00
7	PDA 0.45 cm	3.00, 3.00, 2.00, 2.67, 2.79	3.00, 5.00, 3.00, 2.81, 2.93
8	PDA 0.96 cm	1.00, 2.00, 1.00, 2.00, 2.79	0.50, 2.00, 2.00, 3.00, 2.93
9	PDA 0.47 cm	2.50, 3.00, 2.50, 2.00, 2.50	3.00, 2.50, 2.00, 2.50, 3.00

**Table 6:** Comparison of PPI Values Between Healthy and CHD Neonates at Different Postnatal Time Points.

Time Point (h)	Healthy Group	CHD Group	Between-Group Difference β (95% CI)	*p* Value
	PPI (Mean ± SD)	PPI (Mean ± SD)		
6	2.25 ± 0.03	2.61 ± 0.09	0.36 (−0.07–0.80)	0.0996
12	2.55 ± 0.03	2.22 ± 0.09	−0.63 (−1.10–−0.17)	0.0078 *
24	2.63 ± 0.03	2.25 ± 0.09	−0.64 (−1.11–−0.18)	<0.001 *
48	2.64 ± 0.03	2.31 ± 0.09	−0.58 (−1.05–−0.12)	0.0135 *
72	2.79 ± 0.03	2.46 ± 0.09	−0.53 (−1.10–0.04)	0.0706

* The difference between the groups was statistically significant (*p* < 0.05).

## Data Availability

The data generated in the present study are available from the corresponding author upon reasonable request.

## Abbreviations

The following abbreviations are used in this manuscript:

PPI	Peripheral perfusion index
NCHD	Neonatal congenital heart disease
GAMLSS	Generalized additive models for location, scale and shape
GEE	Generalized estimating equations
